# Comparison of paravertebral, thoracolumbar interfascial plane, and retrolaminar blocks for postoperative analgesia in lumbar disc herniation surgery: A randomized controlled trial

**DOI:** 10.1097/MD.0000000000047141

**Published:** 2026-01-09

**Authors:** Hakan Gokalp Tas, Fethi Akyol, Mustafa Somunkiran

**Affiliations:** a Department of Anesthesiology and Reanimation, Faculty of Medicine, Erzincan Binali Yildirim University, Erzincan, Turkey.

**Keywords:** analgesics, interfascial plane blocks, lumbar vertebrae, nerve block, opioid, postoperative pain

## Abstract

**Background:**

The goal of this prospective randomized controlled experiment was comparing the analgesic efficacy, opioid needs, and adverse effect profiles of paravertebral (PVB) block, thoracolumbar interfascial plane (TLIP) block, and retrolaminar (RLB) block in patients undergoing lumbar disc herniation surgery.

**Methods:**

One hundred eighty-five adults (American Society of Anesthesiologists I–III) undergoing elective lumbar disc herniation surgery made up this single-center study. Among the exclusion criteria were coagulation problems, injection site infection, allergy to local anesthetics, and incapacity to provide informed consent. Patients were randomly assigned into 4 groups: control (systemic analgesia only), PVB, TLIP, and RLB (45 patients per group). An expert anesthesiologist supervised the ultrasonography during each block. The visual analog scale was used to measure postoperative pain at 0, 1, 2, 6, 12, and 24 hours after surgery. This was the primary outcome. Motor block incidence, rescue morphine consumption, and quality of recovery-40 scores at 24 hours were secondary objectives. Unfavorable incidents were noted. Analysis of variance and Kruskal–Wallis tests (*P* < .05) were used to examine the data.

**Results:**

At every time point, the TLIP and RLB groups’ visual analog scale scores were significantly lower than those of the PVB and control groups (*P* < .001). The TLIP and RLB groups consumed considerably less rescue morphine (*P* < .001). The TLIP and RLB groups had the highest quality of recovery-40 scores (*P* < .001). The PVB group had the highest incidence of motor block (26.7%), whereas the RLB group had the lowest rate (8.9%). There were no significant adverse effects noted.

**Conclusion:**

Compared to PVB and control group, TLIP and RLB provide better and longer postoperative analgesia, smaller opioid needs, and higher-quality recovery. Their importance in improved recovery regimens following lumbar disc herniation surgery is supported by their good safety profile.

## 1. Introduction

With an annual frequency of 10% to 15% among healthy adults, low back pain is a significant global public health concern. For low back pain, the typical visual analog scale (VAS) score is approximately 4, indicating moderate pain severity.^[1]^ A common cause of back and leg pain is intervertebral disc herniation, which causes throbbing anguish in the lower extremities along with radicular pain. Lumbar disc surgery is a common procedure for people with moderate to severe back pain and radicular symptoms. Surgery may be necessary for people with chronic symptoms, neurological deficits, or inadequate pain relief with conservative treatment, even though acute intervertebral disc herniation often resolves on its own without surgery.^[1,2]^

After lumbar disc herniation surgery, postoperative pain management is still crucial since inadequate pain management can lead to prolonged hospital stays, slower recovery, and a poorer quality of life.^[3,4]^ Numerous strategies have been used to manage postoperative pain, such as regional anesthetic procedures, epidural steroid injections,^[3]^ nonsteroidal anti-inflammatory drugs (NSAIDs),^[2]^ and even unconventional methods like electroacupuncture.^[5]^ Due of their capacity to provide targeted pain relief while minimizing systemic effects, regional anesthetic procedures have become increasingly popular among these.

In lumbar surgery, the paravertebral block (PVB) is a common regional anesthetic technique. A local anesthetic is injected close to the spinal nerves, and it typically relieves pain over 4 dermatomes (2 above and 2 below the injection site). Patients with spinal anomalies or those for whom neuraxial anesthesia is contraindicated have been shown to benefit greatly from PVB. Additionally, it differs from other peripheral nerve blocks in its ability to reduce visceral and somatic pain.^[6]^ However, PVB requires technical know-how and might have negative effects like vascular injury and pneumothorax, particularly when performed by unskilled professionals.^[7]^

PVB is being replaced by more recent truncal block methods such retrolaminar blocks (RLB) and the thoracolumbar interfascial plane (TLIP). For effective analgesia during lumbar spine surgery, the TLIP block targets the thoracolumbar fascia. According to recent studies, the TLIP block can enhance recovery quality, decrease postoperative pain levels, and lessen the need for rescue medications.^[8]^ In a similar vein, the RLB, which involves administering a local anesthetic into the retrolaminar area, has been associated with shorter recovery periods, reduced postoperative pain scores, and decreased narcotic usage.^[9]^ Compared to PVB, these contemporary techniques are less invasive, simpler to do under ultrasound guidance, and possibly less problematic.

Comparative research is required to evaluate the effectiveness, safety, and viability of TLIP and RLB in the setting of lumbar disc herniation surgery, given their increasing popularity as PVB substitutes. Previous research has primarily focused on the individual efficacy of these methods; however, head-to-head comparisons remain limited. The aim of this study is to investigate at the analgesic efficacy, sensory coverage, and side effect profiles of PVB, TLIP, and RLB in patients having lumbar disc herniation surgery. By doing so, we intend to determine if TLIP and RLB are viable alternatives to PVB, particularly in terms of side effects and patient recovery.

## 2. Materials and methods

The study was conducted in the operating room at Erzincan Mengücek Gazi Education and Research Hospital as a prospective randomized controlled trial. This study was approved by the Erzincan Binali Yildirim University Clinical Research Ethics Committee (Approval No. 2024-07/06, Date: 02/05/2024). All procedures performed were in accordance with the ethical standards of the institutional research committee and with the 1964 Helsinki Declaration and its later amendments. Written informed consent was obtained from all patients prior to participation. From June 2024 to December 2024, the study was carried out.

**Figure 1.**
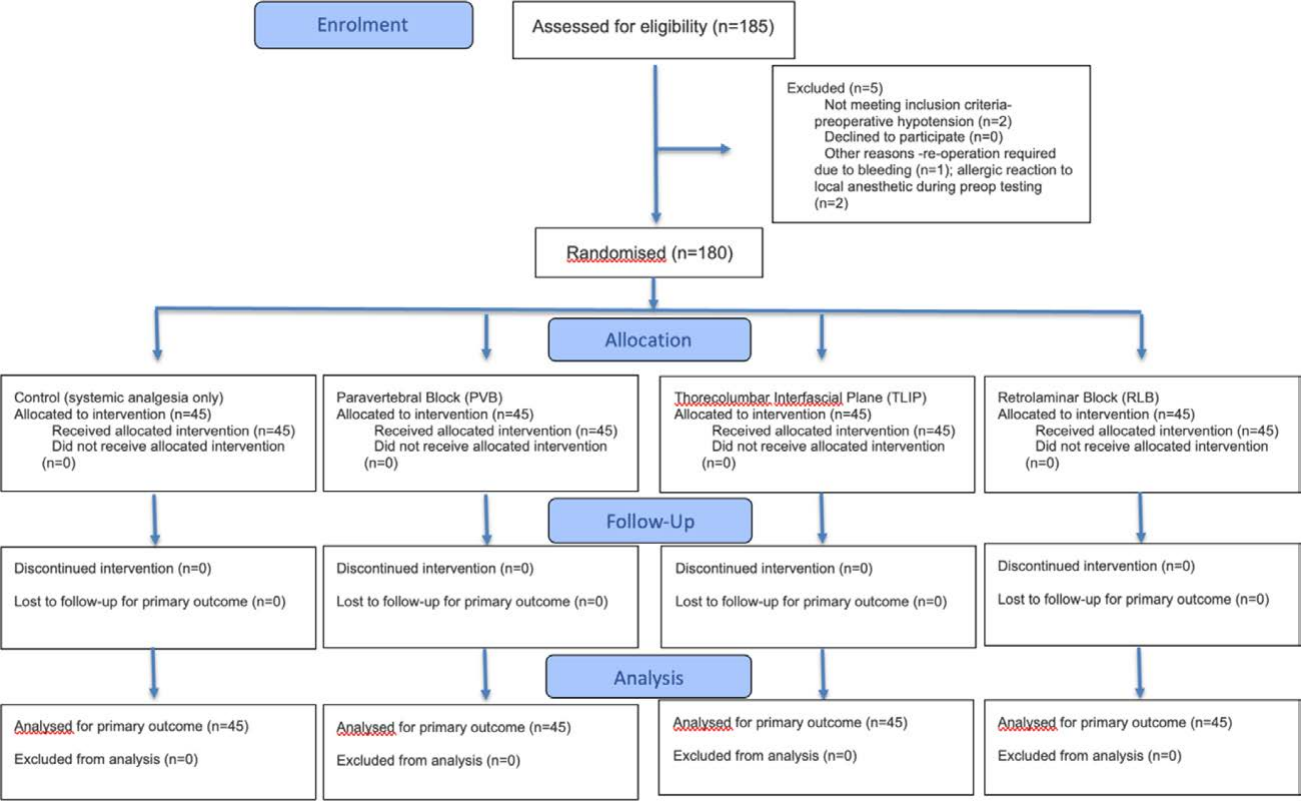
CONSORT flow diagram of the study. CONSORT = Consolidated Standards of Reporting Trials, PVB = paravertebral block, RLB = retrolaminar block, TLIP = thoracolumbar interfascial plane.

The entire inquiry was planned and carried out in accordance with the Consolidated Standards of Reporting Trials criteria. Figure 1 displays the study’s flow diagram.

### 2.1. Trial registration

This study, titled “Comparison of Paravertebral, Thoracolumbar Interfascial Plane, and Retrolaminar Blocks for Postoperative Analgesia in Lumbar Disc Herniation Surgery: A Randomized Controlled Trial,” was registered at ClinicalTrials.gov (Identifier: NCT06933641) prior to patient enrollment, in accordance with ICMJE recommendations.

### 2.2. Participants

One hundred eighty-five patients (based on G-Power analysis) between the ages of 18 and 65 who were having elective lumbar disc herniation surgery and had American Society of Anesthesiologists I–III ratings made up the study. The study excluded patients having a history of opioid dependency, coagulation issues, difficult behavior, pharmaceutical allergies, prior spinal surgery, or unwillingness to participate in the experiment. Two patients were excluded from the study because of preoperative hypotension that occurred before surgery, 1 patient had to have surgery again because of bleeding, and 2 patients had allergic reactions to local anesthetics that were resolved with a simple antihistamine treatment. The trial was completed with 180 patients in total.

### 2.3. Randomization and allocation concealment

IBM SPSS Statistics version [22.0] was used for randomization, and a computer-generated sequence was used. Block randomization was used to allocate an equal number of patients (45 patients each) to the control group (CG group), PVB group, thoracolumbar interfascial plane block (TLIP group), and RLB group. To ensure allocation concealment, a sequentially numbered, sealed opaque envelope strategy was adopted, which meant that neither participants nor investigators were aware of the assigned group until the intervention was performed.

### 2.4. Interventions

After being informed about the study protocol and the operations that would be performed, all patients gave their written consent 1 day before the surgery. The patients were brought to the operating room on the day of the surgery, and venous access was established in the left antecubital region. Peripheral oxygen saturation (SpO_2_), 3-channel electrocardiography, and noninvasive blood pressure monitoring were performed.

Anesthesia was induced using 0.6 mg/kg Rocuronium bromide (Esmeron®, Merck Sharp & Dohme Corp., Kenilworth, NJ, USA), 1 mcg/kg Fentanyl citrate (Fentanyl Citrate®, Johnson & Johnson, New Brunswick, NJ, USA), and 2 mg/kg Propofol (Propofol-Lipuro®, B. Braun Melsungen AG, Melsungen, Hessen, Germany), and sample size calculation was performed using G*Power software (version 3.1.9.7; Heinrich Heine University Düsseldorf, Düsseldorf, North Rhine-Westphalia, Germany). For postoperative pain management after intubation, regional analgesia was administered to all groups (paravertebral, TLIP, and RLBs) with the exception of the control group. About 20 cc of local anesthetic solution, comprising 10 cc of 0.5% bupivacaine and 10 cc of 0.9% NaCl bilaterally, was injected into each side once, with the exception of the control group. The anesthesia was maintained with a mix of sevoflurane, nitrous oxide, and oxygen.

Under the supervision of ultrasound, a single anesthesiologist with twenty-one years of expertise performed all regional blocks. Block success was confirmed by sensory testing in the early postoperative period after extubation. Rescue morphine (3 mg intravenous) was administered for extra analgesia to individuals who scored 8 or higher on the VAS during the postoperative phase.

All patients received intravenous analgesia with 1 g paracetamol and 1 mg/kg tramadol hydrochloride every 12 hours during postoperative ward follow-ups.

### 2.5. Outcome measures

A postoperative pain assessment using the VAS at different times was the main result of this investigation. The quality of recovery-40 (QoR-40) questionnaire, overall further opioid use, and adverse event occurrence were secondary outcomes.

At the first, second, sixth, twelfth, and twenty-fourth hours of ward follow-up, measurements were made of the motor block development, sensory level, and VAS scores. Motor block was assessed at each time point using the modified bromage scale (0 = full movement, 1 = inability to raise extended leg, 2 = inability to flex knee, 3 = inability to move leg or foot). The QoR-40 questionnaire was filled out by every patient 24 hours after surgery.

### 2.6. Statistical analysis

The IBM SPSS 22.0 software was used to conduct the statistical analysis (SPSS Inc., Chicago). The normality assumption was evaluated using the Kolmogorov–Smirnov test. The homogeneity of variance was assessed using the Levene test. The mean ± standard deviation for normally distributed data, the median (min–max) for non-normally distributed variables, and the frequency (%) for categorical variables were used to report descriptive statistics.

The Kruskal–Wallis test was applied to variables that did not follow a normal distribution, whereas the analysis of variance test was employed to compare mean values between groups. The Tukey test for parametric data and the Dunn–Bonferroni test for nonparametric data were used for multiple comparisons.

If the normality conditions were satisfied, correlations between continuous variables were analyzed using the Pearson correlation test; if not, the Spearman correlation test was used.

Data analysis was restricted to patients who submitted complete follow-up data, and patients who were unable to complete follow-up examinations were removed from the final analysis.

All analyses were deemed statistically significant if the *P*-value was <.05.

Sample size: The G-Power 3.1.9.7 software was used to determine the sample size. The minimal number of patients required to provide pertinent data was established because there was no prior research on this particular comparison in the literature. Based on an expected effect size of Cohen’s *d* = 0.25, type-1 error (α) = 0.05, and power (1 − β) = 0.80, 180 participants were required. The main outcome measure used to estimate sample size was the VAS score. To provide sufficient power to detect differences across groups, each group consisted of 45 participants.

## 3. Results

The final analysis comprised 180 patients, evenly split into 4 groups: thoracolumbar interfascial plane block group (TLIP, n = 45), paravertebral block group (PVB, n = 45), retrolaminar block group (RLB, n = 45), and control group (CG, n = 45). There were no discernible variations between the groups in terms of demographic information, such as the distribution of age and gender (*P* > .05).

### 3.1. Postoperative pain assessment

At every time point assessed, VAS scores revealed significant differences between the groups (*P* < .001).

Early postoperative period (0–6 hours): The CG group had the highest VAS scores at the 0- and 1-hour measures (VAS 0 hour: 6.1 ± 1.3, VAS 1 hour: 6.3 ± 1.2), suggesting that patients without regional anesthetic had much higher pain levels right after surgery. The PVB group outperformed CG in terms of pain alleviation among the block groups, although it was still below TLIP and RLB. The TLIP and RLB groups had the lowest VAS values at these early time points, with RLB showing somewhat higher analgesic efficacy (VAS 0 hour: 3.1 ± 1.0, VAS 1 hour: 3.3 ± 0.9), indicating a quick commencement of action.

Intermediate period (2–12 hours): Significant differences remained by the 2-hour mark (*P* < .001), with the TLIP and RLB groups continuing to reduce pain better than PVB (VAS 2h: PVB: 4.1 ± 1.0, TLIP: 3.2 ± 0.9, RLB: 3.0 ± 0.8). TLIP and RLB continued to be better at 6 and 12 hours, even though all groups’ VAS scores marginally increased. At 12 hours, the PVB group’s VAS scores were notably closer to the CG group’s (VAS 12h: CG: 5.5 ± 1.2, PVB: 4.3 ± 1.1, TLIP: 3.4 ± 1.0, RLB: 3.1 ± 0.9), suggesting that PVB’s analgesic impact gradually diminished in comparison to TLIP and RLB.

Late period (24 hours): CG maintained the highest pain levels (VAS 24 hours: 5.1 ± 1.3) until the 24th hour, whereas the RLB group had the lowest pain scores (VAS 24 hours: 2.9 ± 1.2), closely followed by TLIP (VAS 24 hours: 3.1 ± 1.0). PVB provided moderate pain alleviation (VAS 24 hours: 3.9 ± 1.1), but was less effective than TLIP and RLB.

**Figure 2.**
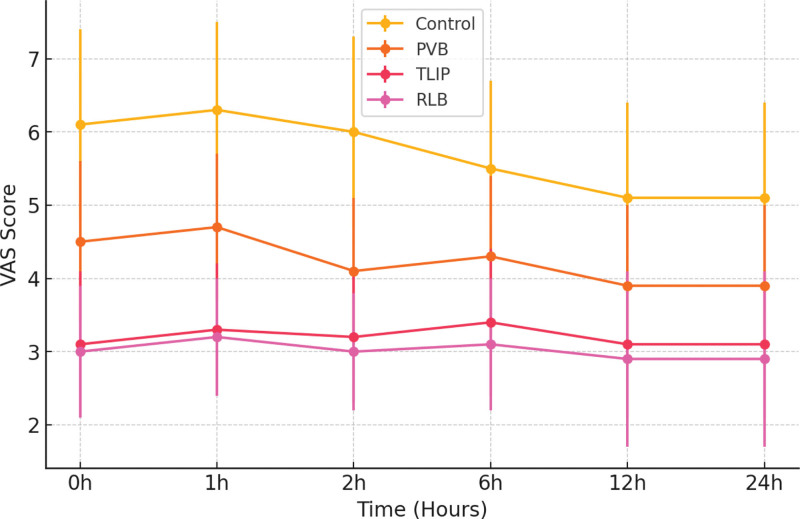
VAS scores over time across groups. PVB = paravertebral block, RLB = retrolaminar block, TLIP = thoracolumbar interfascial plane, VAS = visual analog scale.

These data show that both TLIP and RLB provide better early and prolonged analgesia, with RLB providing the most consistent pain reduction over time. VAS scores over time across groups are shown in Figure 2.

### 3.2. Rescue morphine requirement

**Figure 3.**
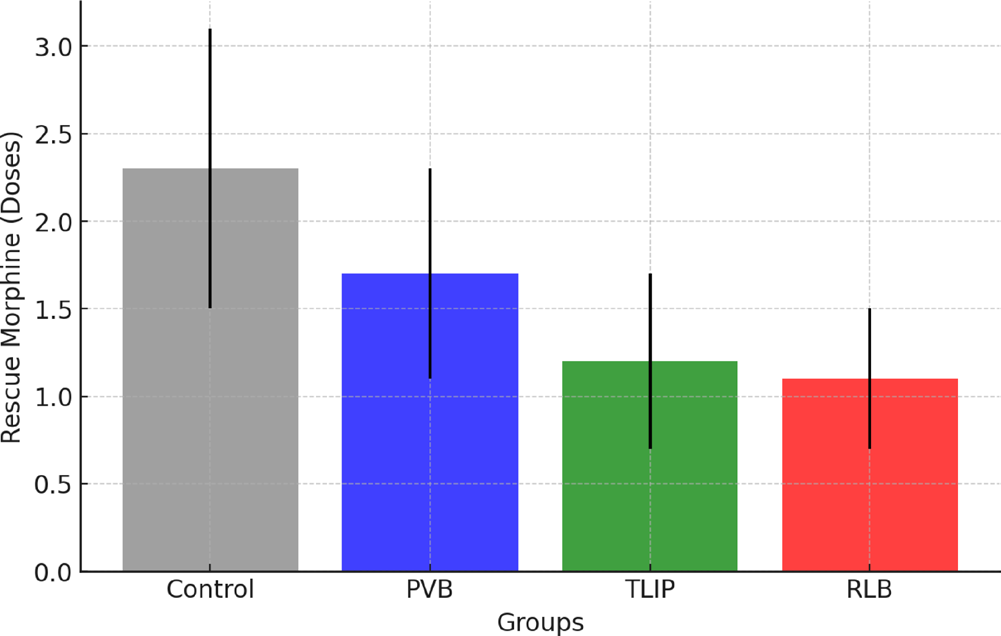
Rescue morphine requirement across groups. PVB = paravertebral block, RLB = retrolaminar block, TLIP = thoracolumbar interfascial plane.

The need for rescue morphine varied considerably across groups (*P* < .001). The CG group received the most rescue morphine (2.3 ± 0.8 doses), followed by PVB (1.7 ± 0.6 doses), while the TLIP and RLB groups required the least (1.2 ± 0.5 and 1.1 ± 0.4 doses, respectively). This confirms TLIP and RLB’s better analgesic efficacy over PVB. Rescue morphine requirement across groups is shown in Figure 3.

### 3.3. Quality of recovery

**Figure 4.**
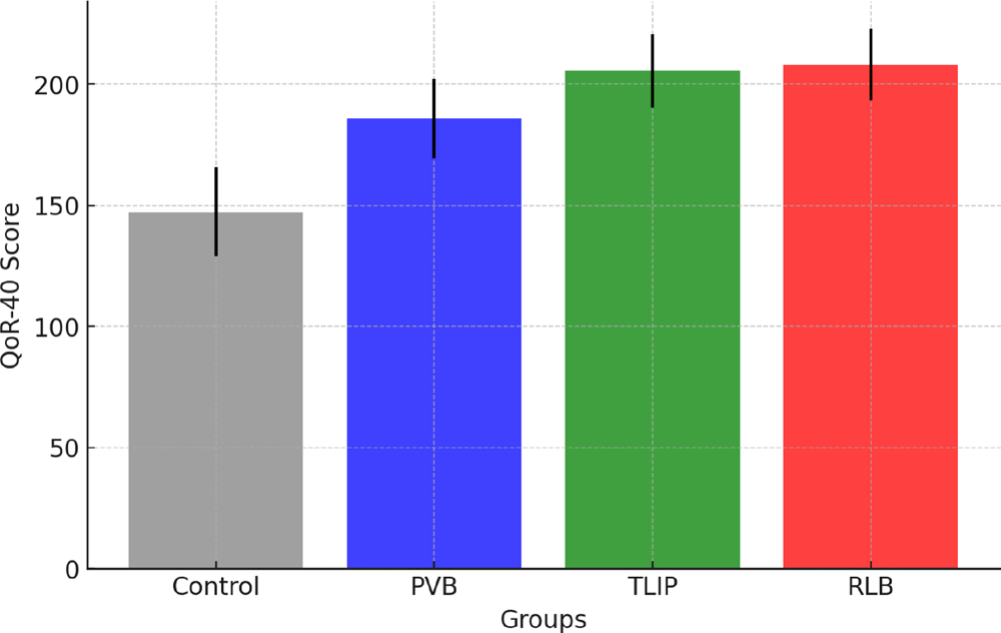
Quality of recovery (QoR-40) scores across groups. PVB = paravertebral block, QoR = quality of recovery, RLB = retrolaminar block, TLIP = thoracolumbar interfascial plane.

The Quality of Recovery-40 (QoR-40) scores differed considerably between groups (*P* < .001). The TLIP and RLB groups had the highest QoR-40 ratings (205.6 ± 15.2 and 208.1 ± 14.8, respectively), while the CG group had the lowest (147.3 ± 18.5). The PVB group was not as good as TLIP and RLB, but they did demonstrate an intermediate improvement (185.7 ± 16.4). Quality Of Recovery (QoR-40) scores across groups are shown in Figure 4.

### 3.4. Motor block incidence

**Figure 5.**
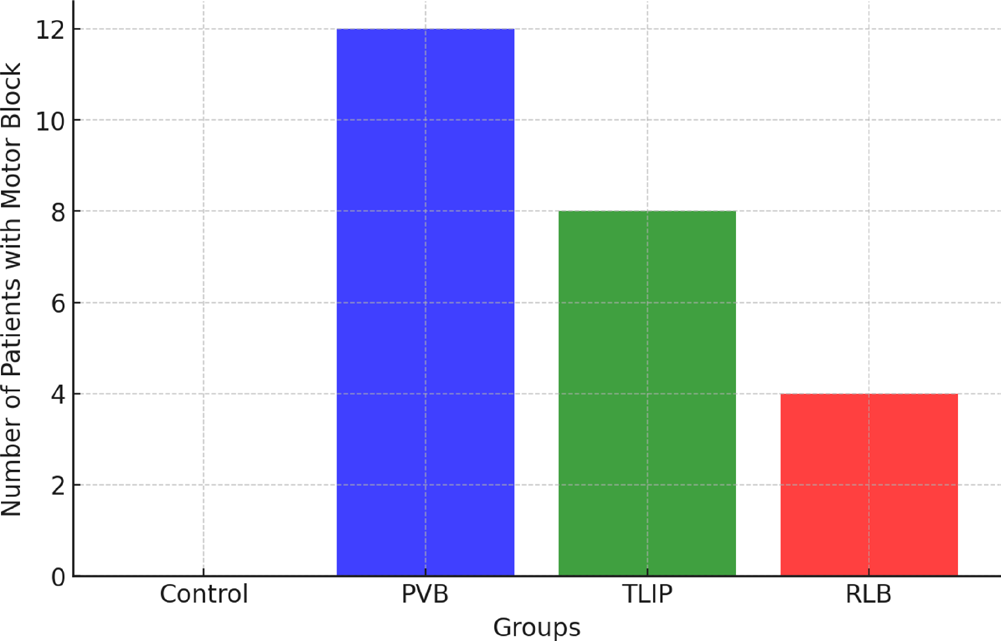
Motor block incidence across groups. PVB = paravertebral block, RLB = retrolaminar block, TLIP = thoracolumbar interfascial plane.

There was a significant difference in the incidence of motor block formation between the groups (*P* < .001). Motor block was absent in the control group but was seen in 12 patients (26.7%) in the PVB group, 8 patients (17.8%) in the TLIP group, and 4 patients (8.9%) in the RLB group. The RLB group had a decreased incidence of motor block, which implies that it may minimize motor impairment and offer adequate analgesia, making it a better choice for early mobilization and rehabilitation. Motor block incidence across groups is shown in Figure 5.

### 3.5. Surgical duration

The use of regional blocks did not appear to lengthen the surgical procedure, as there were not significant differences in the duration of the surgery between groups (*P* = .78).

### 3.6. Summary

According to the results, TLIP and RLB outperform PVB in terms of postoperative analgesia, opioid usage, and recovery quality. RLB had the best pain management in the first 6 hours after surgery, and both TLIP and RLB continued to have better analgesic effects for the full twenty-four hours. The RLB group had the lowest incidence of motor block, suggesting that it is a feasible option to PVB for lumbar disc herniation surgery. Further research is needed to evaluate long-term outcomes and patient satisfaction.

## 4. Discussion

Effective postoperative pain management after lumbar disc herniation surgery is critical for faster healing, earlier mobilization, and patient satisfaction. This prospective randomized controlled trial compared the postoperative analgesic efficacy and side effect profiles of PVB, TLIP block, and RLB in patients undergoing lumbar disc surgery. Our data show that TLIP and RLB provided better pain relief, reduced opioid intake, and enhanced functional recovery than PVB and routine systemic analgesia. These findings add to the expanding body of information supporting interfascial plane blocks as valuable components of multimodal analgesic methods in spine surgery.

PVB has long been employed for thoracic and lumbar surgeries because it provides consistent somatic and visceral analgesia coverage. Studies consistently reveal that PVB lowers postoperative pain and narcotic consumption in thoracic and abdominal procedures.^[10]^ However, the efficacy of PVB in lumbar spine surgery is less well-known. In our study, PVB produced superior analgesia than systemic analgesia alone, although its efficacy decreased with time, particularly after 12 hours. This is consistent with previous research indicating that the local anesthetic dissemination in the paravertebral area may be variable at the lumbar level because of anatomical variances.^[11]^

Newer interfascial plane blocks, such as TLIP and RLB, have emerged as attractive alternatives because to their ease of use, ultrasound-guided precision, and low risk of complications. Interfascial plane blocks were first used in clinical practice to provide analgesia during thoracic and abdominal procedures, but their use has now expanded to include spine surgery. El-Boghdadly et al highlighted that these blocks, including TLIP and RLB, offer effective pain relief with minimal motor involvement, making them particularly useful for spine procedures.^[12]^ TLIP focuses on the thoracolumbar fascia, which is highly innervated and plays an important role in posterior lumbar pain transmission. Recent randomized trials have revealed that TLIP is effective in lowering postoperative pain scores and narcotic usage.^[13]^ Our findings, which suggest that TLIP provides prolonged analgesic effect over 24 hours, are similar with previous publications.

RLB, which was originally touted as a simpler alternative to thoracic PVB, has since become increasingly common in spine surgery. It is technically easier to execute, and multiple studies have emphasized its positive safety profile.^[14]^ In our study, RLB had the lowest VAS scores and the lowest incidence of motor block, which could increase its utility in enhanced recovery pathways. RLB’s greater performance is most likely owing to its ability to provide consistent local anesthetic spread over the posterior thoracolumbar plane, providing long-term sensory coverage while without immediately affecting motor neurons.

The time-segmented examination of VAS scores in our study sheds light on the relative efficacy of these blocks during the perioperative period. TLIP and RLB produced rapid-onset analgesia in the early postoperative period (0–6 hours), exceeding both the PVB and control groups. This pattern parallels prior studies that show that interfascial plane blocks are associated with rapid local anesthetic distribution and early sensory blockade.^[15]^

In the intermediate postoperative period (6–12 hours), TLIP and RLB continued to provide superior analgesia, but PVB’s efficacy gradually declined. This decrease is consistent with earlier studies indicating that local anesthetic distribution in the paravertebral region is less predictable at lumbar levels.^[15,16]^ By 24 hours postoperatively, TLIP and RLB remained considerably superior than PVB and systemic analgesia, showing their efficacy for long-term postoperative pain control.

To prevent opioid-related side effects such as nausea, vomiting, drowsiness, ileus, and respiratory depression, current postoperative pain treatment prioritizes opioid intake reduction. The TLIP and RLB groups required far fewer rescue opioid doses than the PVB and control groups. This observation is consistent with the emerging evidence that interfascial plane blocks are efficient opioid-sparing methods.^[15,17]^ Enhanced opioid-sparing benefits are especially important in spine surgery groups, which may include patients at high risk for opioid-related problems, such as those with obesity or obstructive sleep apnea.

The quality of recovery-40 (QoR-40) questionnaire assesses postoperative recovery in multiple dimensions, including physical comfort, emotional well-being, and functional independence. The finding that the TLIP and RLB groups had the highest QoR-40 scores is consistent with previous enhanced recovery after surgery (ERAS) literature, which underscores the role of efficient regional analgesia in promoting early mobilization and better patient satisfaction.^[18,19]^ The low incidence of motor block in the RLB group likely contributed to greater mobility and functional recovery. This is consistent with prior research that reported decreased motor impairment with interfascial plane blocks compared to neuraxial or paravertebral approaches.^[20]^

All blocks were administered under ultrasound supervision by an experienced anesthesiologist, which likely contributed to our study’s low complication rate. Nonetheless, PVB was associated with the highest incidence of motor block, perhaps limiting its use in ERAS pathways. This discovery matches previous worries about the unpredictable spread to motor roots with lumbar PVB.^[16]^ In contrast, both TLIP and RLB demonstrated lower rates of motor block, indicating their suitability for application in procedures where motor function must be preserved.

Despite these promising results, it is crucial to note that interfascial plane blocks are not without danger. Local anesthetic systemic toxicity (LAST) and accidental intravascular injection have been documented in thoracic block case reports, highlighting the necessity of accurate injection technique, appropriate dosage, and ongoing hemodynamic monitoring.^[21]^

There are various limitations on this study. Due to its single-center design, external validity might be constrained. Furthermore, evaluation of longer-term outcomes, such as persistent postoperative pain, which may be clinically significant, is not possible within the 24-hour follow-up period. The fact that just 1 skilled anesthesiologist performed each block limited the study’s applicability to facilities with different degrees of procedural experience. Lastly, information on patient satisfaction and sleep quality, which could have offered useful supplementary end measures, was not gathered.

## 5. Conclusion and future directions

The use of TLIP and RLB for postoperative analgesia following lumbar disc herniation surgery is well supported by our findings. Compared to PVB and systemic analgesia, both blocks provide better pain relief, less opioid needs, and higher-quality recovery. TLIP and RLB are especially well-suited for inclusion in enhanced recovery pathways due to their good safety profile and decreased incidence of motor block. To further improve the best analgesic techniques for lumbar spine surgery, multicenter trials with longer follow-up and more patient-centered outcomes are essential.

## Acknowledgments

The authors thanks all thi physicians contributed the study

## Author contributions

**Conceptualization:** Hakan Gokalp Tas, Mustafa Somunkiran.

**Data curation:** Hakan Gokalp Tas.

**Formal analysis:** Hakan Gokalp Tas.

**Investigation:** Hakan Gokalp Tas, Fethi Akyol, Mustafa Somunkiran.

**Methodology:** Hakan Gokalp Tas.

**Project administration:** Mustafa Somunkiran.

**Resources:** Fethi Akyol.

**Software:** Fethi Akyol.

**Supervision:** Hakan Gokalp Tas, Fethi Akyol.

**Validation:** Hakan Gokalp Tas.

**Writing – original draft:** Hakan Gokalp Tas.

**Writing – review & editing:** Fethi Akyol.
